# Nitric oxide enhancement strategies

**DOI:** 10.4155/FSO.15.48

**Published:** 2015-08-01

**Authors:** Nathan S Bryan

**Affiliations:** 1 Department of Molecular & Human Genetics, Baylor College of Medicine, 1 Baylor Plaza, Houston, TX 77030, USA

**Keywords:** guanylyl cyclase, nanotechnology, nitrate, nitric oxide, nitrite, nitrosothiols

## Abstract

It is becoming increasingly clear that many diseases are characterized or associated with perturbations in nitric oxide (NO) production/signaling. Therapeutics or strategies designed to restore normal NO homeostasis will likely have broad application and utility. This highly complex and multistep pathway for NO production and subsequent target activation provides many steps in the endogenous pathway that may be useful targets for drug development for cardiovascular disease, antimicrobial, cancer, wound healing, etc. This article will summarize known strategies that are currently available or in development for enhancing NO production or availability in the human body. Each strategy will be discussed including exogenous sources of NO, use of precursors to promote NO production and downstream pathways affected by NO production with advantages and disadvantages highlighted for each. Development of NO-based therapeutics is and will continue to be a major focus of biotech, academia as well as pharmaceutical companies. Application of safe and effective strategies will certainly transform health and disease.

**Figure 1.**
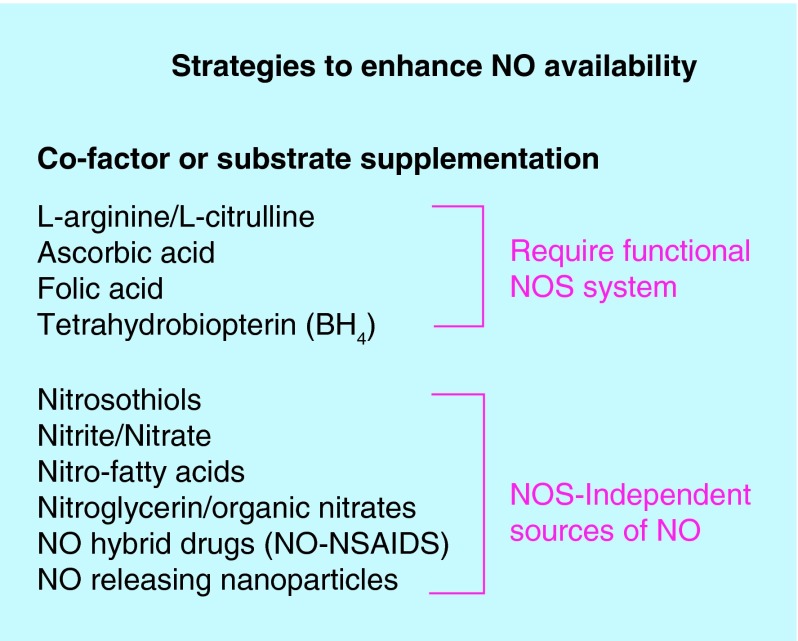
**Viable strategies to enhance nitric oxide production/availability.** NO: Nitric oxide; NOS: Nitric oxide synthase.

The discovery of the NO pathway is recognized as a critical advancement in cell signaling and has led to major advancements in many clinical areas that have the ability to transform biotechnology and medicine. The discovery of NO and its function has been said is one of the most important in the history of cardiovascular medicine. The Nobel Prize in Physiology or Medicine was awarded in 1998 to its discoverers, Drs Louis J Ignarro, Robert Furchgott and Ferid Murad. The Nobel committee summarized the award as follows: “The signal transmission by a gas that is produced by one cell, penetrates through membranes and regulates the function of another cell, represents an entirely new principle for signaling in biological systems.” Therefore safe and effective strategies to replete or recapitulate endogenous NO production or availability will have enormous impact on public health and disease prevention. More than 15 years after the Nobel Prize was awarded for the discovery of NO and after more than 130,000 published scientific and medical papers, there is still a lot to be learned about its production and regulation and all of its biological functions. This article will summarize contemporary technologies for delivering bioactive NO in the human body.

Continuous and regulated generation of NO is essential for the health of the cardiovascular system, immune and nervous system. Decreased production and/or bioavailability of NO is recognized as being one of the earliest events in the onset and progression of many diseases. The production of NO from L-arginine by nitric oxide synthase (NOS) enzymes is one of the most complicated and complex reaction in the body involving a 5-electron oxidation with many cofactors and prosthetic groups. As a result, there are many steps in the pathway that may be affected that ultimately lead to decreased or no NO production. Once produced, with a half-life of approximately 1 s, NO can be quickly scavenged further reducing its half-life and not allowing it to perform its actions. It is, therefore, a war of attrition when it comes to producing bioactive NO, and is a remarkable feat that this short-lived gas is responsible for so many essential cellular activities. Loss of NO production, termed endothelial dysfunction is recognized as one of the earliest events in the onset and progression of chronic disease. Recently, the rescue pathway for loss of constitutive NO production from NOS enzymes has been discovered through the serial reduction of endogenous and/or dietary nitrate and nitrite to NO [1].

NO is recognized by the scientific and medical community as one of the most important molecules produced within the body, yet there are currently only three US FDA approved products on the market directly related to NO: organic nitrates, such as nitroglycerin for the treatment of acute angina; inhaled NO therapy for neonates for treatment of pulmonary hypertension; and phosphodiesterase inhibitors which do not affect NO production but act to prevent the breakdown of the downstream second messenger of NO, cyclic guanosine monophosphate (cGMP). With the knowledge gained in the physiology and pharmacology of NO, better and new drugs as well as NO delivery systems are being designed for many contemporary diseases and medical problems.

There are a number of NO-based therapies in development, including technologies designed to activate and promote NO synthesis from NOS, NO donating compounds, therapies designed to modulate post-translational protein modifications through S-nitrosation and therapies designed to affect or prolong downstream signaling pathways from NO. There are also many dietary supplements and nutraceuticals marketed toward enhancing NO. The method of delivery of NO and cellular and molecular specificity is of utmost importance. The subject matter of this article will focus only on authentic NO gas enhancement strategies and not address downstream targets. A substantial knowledge of the NO signaling pathway has been gained during the past three decades. The current published literature of NO-based research will be discussed and some of the potential NO enhancement strategies for drug and/or device development.

## Market opportunities

In the worldwide pharmaceutical market, share of drugs where NO is involved in the mechanism of action was US$58 billion in 2009 and rose to US$102 billion in 2014 and expected to reach US$147 billion in 2019 as new drugs with NO-based mechanisms are introduced into the market [2]. In fact, most large pharmaceutical companies have a division committed to NO-based therapies and many new start-ups are founded upon intellectual property around NO.

### Strategies to restore or enhance NO

There are finite ways to safely and effectively enhance or restore NO availability in the human body. These are highlighted in Figure 1. Part of these strategies target NOS enzymes as a means to enhance NO production and other strategies provide NO as an exogenous NOS-independent source of NO. Since most chronic diseases are characterized, or at least associated with dysfunctional NOS, NOS-dependent strategies utilizing L-arginine and/or L-citrulline have proven largely ineffective [3,4]. This article will focus primarily on NOS-independent strategies.

A better understanding of the processes leading to cardiovascular disease is allowing for adoption of new principles of therapy that may be more beneficial for patients. Gaseous NO, produced within endothelial cells or delivered exogenously diffuses across the endothelial cell and into the underlying smooth muscle cell, where it activates soluble guanylyl cyclase (sGC) to produce vasodilatation. Other examples are in the immune system and nervous system. Therefore the basis for providing an exogenous source of NO will allow NO to then activate its downstream targets and provide rescue to the cells or tissues.

## Delivery of NO

Targeted delivery of NO at precise cellular locations poses an extreme challenge with respect to recapitulating physiological production of NO. There are several methods of delivery of NO. The most common and effective for targeted delivery to the pulmonary circulation is inhaled NO. There are also biomaterials being developed for sustained release of NO for topical applications for wound healing and infections, among others. Nanoparticle delivery of NO is an emerging field, particularly in cancer biology. NO-eluting stents or NO coating of orthopedic implants for preventing biofilm growth and infection is an area of active development. There are a number of NO releasing or generating materials being developed covered in other articles in this issue, but controlled and site-specific delivery through nanoparticles offers enormous advantage. Nanoparticle delivery of NO shows enormous promise in infections, specifically antibiotic-resistant bacteria. Through targeted and concentration-dependent delivery of NO, these technologies exert antimicrobial activity based largely through formation of reactive nitrogen oxide species (RNOS) intermediates especially at high concentrations (above 1 mM) where they can react with amino acid residues of bacteria proteins. NO and/or RNOS cause direct nitrosative damage to DNA, including causing strand breaks, formation of abasic sites and deamination of cytosine, adenine and guanine. RNOS also cause increased generation of hydrogen peroxide (H_2_O_2_) and alkylating agents, which themselves damage DNA. NO or NO-derived species can inhibit DNA repair enzymes, including DNA alkyl transferases whose cysteine residues are S-nitrosylated. RNOS and/or nitrite also react with prosthetic groups of proteins, like Fe–S clusters and heme [5]. The same is true for targeted delivery of NO to cancer cells whereby NO at the right concentration can kill rapidly dividing cancer cells. Topical application of NO releasing bandages can provide treatment for skin infections and healing of wounds.

## Inhalative NO therapy

NO delivered through inhalation therapy is a selective pulmonary vasodilator and is used and has been used for decades as a safe and effective therapy to increase pulmonary vascular NO and subsequent cGMP concentrations. Inhaled NO causes the pulmonary vasculature to dilate without the side effects of systemic reduction in blood pressure or hypoxemia. As early as the 1990's Zapol and Frostell hypothesized that delivering low concentrations of NO through inhalation would relax pulmonary vascular smooth muscle and cause pulmonary vascular dilation [6]. These investigators thought that the inhaled NO would quickly be inactivated upon reaching the bloodstream, due to oxidation by oxyhemoglobin thereby avoiding the systemic side effects observed with NO-donor molecules or therapies. Inhaled NO therapy has been in widespread clinical use for more than 10 years. However it should be noted that proper methods are required for the safe administration of NO. NO can react with oxygen to form nitrogen dioxide (NO_2_), a toxic air pollutant. Since the amount of NO_2_ formed depends on the concentrations of NO and oxygen and also how long the two gases are mixed together, proper mixing and delivery systems are required. Inadvertent exposure of the lung to NO_2_ can cause significant injury. Since NO is unstable especially when mixed with oxygen, it has to be shipped and stored in nitrogen. During the development of inhaled NO therapy, new equipment had to be developed to ensure reliable, therapeutic levels of NO to be continuously delivered to keep the reaction with oxygen at a minimum. Additional considerations include the requirement that the NO delivery system be portable to enable transport of patients breathing NO. Today there is commercially available equipment which minimizes the time during which oxygen and NO are mixed prior to administration to the patient. Monitors are also necessary for real-time measurement of NO, oxygen and NO_2_ administered to the patient. This technology has been used safely and effective for more than a decade in neonatology. Use of NO inhalative therapy requires awareness of several ‘side effects’ associated with breathing NO. These include methemoglobinemia, inhibition of platelet function and increases in pulmonary capillary wedge pressure (in the setting of LV dysfunction). Clinicians also need to be aware of the rebound pulmonary hypertension that occurs if inhaled NO is abruptly discontinued. There are also extrapulmonary effects of inhalative NO therapy. The discovery of an endocrine function of NO [7] allows for NO bioactivity to be transported throughout the systemic circulation when NO is delivered or generated in a single biological compartment. In fact, inhaled NO protects the heart from injury from heart attack [8].

## Inorganic nitrite

Inorganic nitrite (NO_2_^-^) is known as an undesired residue in the food chain or as inert oxidative end products of endogenous NO metabolism. However, from research performed over the past decade, it is now apparent that nitrite is physiologically recycled in blood and tissues to form NO and other bioactive nitrogen oxides [1]. Thus, nitrite is now recognized as a reservoir of NO-like bioactivity to be acted upon when enzymatic NO production from NOS is insufficient. Nitrite is an oxidative breakdown product of NO that has been shown to serve as an acute marker of NO flux/formation [9]. Nitrite is in steady-state equilibrium with S-nitrosothiols [10] and has been shown to activate soluble guanylyl cycles (sGC) and increase cGMP levels in tissues [10]. Therefore is an ideal candidate for restoring both cGMP dependent and independent NO signaling. Nitrite can be reduced to NO by a variety of enzymes including hemoglobin and xanthine oxidoreductase. Hunter and colleagues reported that inhalation of high concentrations of nebulized nitrite reduced PAP in newborn lambs with hypoxia- and U46619-induced pulmonary vasoconstriction. Additionally, experiments in primates revealed a beneficial effect of long-term application of nitrite on cerebral vasospasm. Topical application of nitrite is an effective treatment for skin infections and ulcerations. Furthermore, in the stomach, nitrite-derived NO plays an important role in host defense and in regulation of gastric mucosal integrity. Oral nitrite has also been shown to reverse L-NAME-induced hypertension and serve as an alternate source of NO *in vivo*. More recently, these investigators reported that intermittent administration of a low-dose nitrite aerosol could attenuate pulmonary vascular remodeling in rodent models of pulmonary arterial hypertension [1].

In addition to the oxidation of NO, nitrite is also derived from reduction of salivary nitrate by commensal bacteria in the mouth and GI tract as well as from dietary sources such as meat, vegetables and drinking water. The metabolic activation of nitrate from dietary or endogenous sources requires its initial reduction to nitrite, and because mammals lack nitrate reductase enzymes, this conversion is dependent upon oral commensal bacteria as well as bacteria in the GI tract and on body surfaces [11]. Human nitrate reduction requires the presence of these bacteria, revealing a functional symbiotic relationship, since mammalian cells cannot effectively metabolize this anion. The salivary nitrate levels can approach 10 mM and nitrite levels 1–2 mM after a dietary nitrate load. When saliva enters the acidic stomach (1–1.5 l per day), much of the nitrite is rapidly protonated to form nitrous acid (HNO_2_; pKa ˜3.3), which decomposes further to form NO and other nitrogen oxides [12].

The discovery of this mammalian nitrogen cycle has led researchers to explore the role of nitrate and nitrite in physiological processes that are known to be regulated by NO. Much of the recent focus on nitrite physiology is due to its ability to be reduced to NO during ischemic or hypoxic events. Nitrite reductase activity in mammalian tissues has been linked to the mitochondrial electron transport system, protonation, deoxyhemoglobin and xanthine oxidase. There are also a number of natural products that have been shown to be oxygen-independent nitrite reducers [13] that can provide an effective system for generating NO from nitrite. Since a substantial portion of steady-state nitrite concentrations in blood and tissue are derived from dietary sources [10], for conditions associated with NO insufficiency, a first line of defense may be provided by modulation of nitrate and/or nitrite intake. Nitrite and nitrate therapy or supplementation may restore NO homeostasis from endothelial dysfunction providing benefit in a number of diseases characterized by NO insufficiency. If so, this will provide the basis for new therapeutic or preventive strategies, and new dietary guidelines for optimal health. Studies using a patented nitrite formulation (US patents 8,303,995, 8,298,589, 8,435,5708 and 8,962,038) marketed as a nutraceutical in the form of an orally disintegrating tablet found that it could modify cardiovascular risk factors in patients over the age of 40, significantly reduce triglycerides and reduce blood pressure [14]. Single administration of this lozenge leads to peak plasma levels of nitrite around 1.5 µM that will sustain for several hours. This same lozenge was used in a pediatric patient with argininosuccinic aciduria and significantly reduced his blood pressure when prescription medications were ineffective [15]. A more recent clinical trial using the NO lozenge reveals that a single lozenge can significantly reduce blood pressure, dilate blood vessels, improve endothelial function and arterial compliance in hypertensive patients [16]. Furthermore in a study of prehypertensive patients (BP >120/80 <139/89 mmHg), administration of one lozenge twice daily leads to a significant reduction in blood pressure (12 mmHg systolic and 6 mmHg diastolic) after 30 days [17]. The same lozenge was used in an exercise study and was found to lead to a significant improvement in exercise performance [18]. These studies clearly demonstrate the safety and efficacy of low doses of nitrite in humans. From a public health perspective, we may be able to provide better dietary recommendations and dramatically affect the severity and incidence of cardiovascular disease and the subsequent clinical events. Nitrite itself may provide therapeutic benefit embedded in certain materials.

## NO donating drugs

NO donors were really the first class of NO-based therapies. NO donors broadly speaking are a heterogeneous group of different chemical classes of compounds that either decompose spontaneously in a pH dependent manner or are metabolized in cells and tissues to generate NO. These different classes of NO donating drugs all have different pharmacodynamic, pharmacokinetic, and toxicological properties. A common feature of all of these compounds is that they can release NO and lead to NO dependent biological effects such as relaxation of blood vessels. Nitrovasodilators, such as nitroglycerin are used in the management of various acute and chronic cardiovascular pathologies. Their pharmacologic effects are mediated biochemically through the release of NO in the vasculature independent of endogenous endothelial NOS (eNOS). Once liberated, NO activates sGC in the smooth muscle, increases the concentrations of the secondary messenger cyclic GMP, alters calcium flux and ultimately causes relaxation. There are a select few nitrovasodilators that are in clinical use today, however, all these drugs have been used in medicine long before the discovery of NO as a biological signaling molecule. Clinically available NO donors approved for use in the US in patients with cardiovascular disease include nitroglycerin (GTN), isosorbide dinitrate (ISDN), isosorbide mononitrate (IS-5N), amyl nitrite, and sodium nitroprusside (SNP). Pentaerythrityltetranitrate (PETN) has been approved for use in the US for many years, but has been largely replaced by ISDN and IS-5N. Nicorandil and molsidomine (which is converted to the active moiety, 3-morpholinosydnonimine [linsidomine, SIN-1], *in vivo*) are not approved for use in the US, but, like PETN, are available abroad. These drugs are administered both orally and transdermally.

Although nitrovasodilators are very effective in acute care situation, the long term use is severely limited by the rapid development of tolerance to their vasodilatory effects. In order for organic nitrates to maintain their vasodilatory effects when used chronically, they must be cycled off for an 8–12-hour period. This interruption complicates organic nitrate therapy in patients who may require around-the-clock angina protection. Interestingly, in contrast to the vasodilatory effects, tolerance development to the platelet anti-aggregatory effects of organic nitrates does not appear to be significant [19]. The mechanisms of nitrate tolerance have been described as a 130-year old mystery [20] but exhibits several major features, namely modifications or depletion of free sulfhydryl groups, reduced organic nitrate metabolism to NO and inactivation of aldehyde dehydrogenase-2 (ALDH2), neurohormonal counter-regulation, presence of withdrawal rebound, presence of vascular oxidative stress, including increased superoxide (O_2_^•−^) production and accumulation, and extensive alteration of gene expression in the aorta. Chronic organic nitrate therapy has been associated with reduced survival when used in patients with coronary artery disease [21] but the underlying mechanisms are still not known. It is clear however that use of organic nitrate therapy leads to increased vascular oxidative stress which in turn can produce endothelial dysfunction. As a result, use of these compounds is limited to acute treatments, such as acute angina or for emergency hypertensive crises.

## Diazeniumdiolates

Diazeniumdiolates, compounds of structure R_1_R_2_NN(O)=NOR_3_, which have also been called NONOates, have proven useful for treating an increasing diversity of medical disorders in relevant animal models [22] but have yet to reach clinical utility in humans. NONOates are generated by exposing various nucleophile compounds to NO gas at a few atmospheres of pressure. The resulting compounds are stable as a solid and highly soluble in aqueous solution, releasing 2 moles of NO per mole of donor compound. Decomposition rates are dependent upon pH, temperature and the chemical characteristics of the donor compound, generating compounds whose NO generation rate can be predicted and adjusted. A distinct advantage of this class of NO donors is that they generate NO spontaneously, without any need for electron transfer, co-factors, metabolic activation, or oxidation-reduction activation. A disadvantage is the spontaneous NO release which presents a challenge for targeted delivery. In order to provide a mechanism for targeted NO release, photosensitive precursors to diazeniumdiolates have been developed. There are three different classes of photo-triggered diazeniumdiolates: 2-nitrobenzyl derivatives, meta-substituted benzyl derivatives, and naphthylmethyl and naphthylallyl derivatives. This photo-triggered diazeniumdiolate derivative may prove useful in future pharmacological investigations. Potential applications include inhibition of restenosis after angioplasty, preparation of thromboresistant medical devices, reversal of vasospasm, and relief of pulmonary hypertension. Beneficial effects of diazeniumdiolate therapy have been reported in a patient with acute respiratory distress syndrome but these compounds have not entered clinical trials in humans as yet.

## NO-hybrid drugs

In the last few years, a revision of the ‘one-compound-one-target’ paradigm has led pharmacologists and pharmaceutical chemists to develop new classes of molecules which combine different pharmacodynamic properties. This innovative strategy has produced hybrid drugs, with a dual mechanism of action that captures its original mechanism along with the slow release of NO. These drugs are synthesized by inserting appropriate NO-donor chemical groups (i.e., nitrate esters, nitrosothiols, etc.), linked to a known parent drug compound. This strategy has opened up the possibility of designing new classes of drugs that are capable of delivering NO into tissues and the bloodstream in a sustained and controlled manner and perhaps offset many of the unwanted side effects. The approach has led to the synthesis of several new chemical entities whose pharmacologic profile provides a better safety profile than the parent drug. These hybrids offer the advantage of combining a basic mechanism of action from the parent drug (e.g., cyclooxygenase inhibition, β-antagonism or ACE inhibition) with a slow release of NO, which may be useful either to reduce adverse side effects (e.g., the gastrotoxicity of NSAIDs) or to improve the effectiveness of the drug (e.g., conferring direct vasorelaxing and antiplatelet effects on an ACE inhibitor). Leading this charge are the NO-NSAIDS. NSAIDs, including those that are selective for cyclooxygenase-2, were among the most widely used drugs until they were recalled by the FDA due to an increased risk of serious cardiovascular events, including heart attacks and strokes. It was thought that the new NO-NSAIDS could be developed to offer significant advantages over conventional and Cox-2-selective NSAIDs and prevent many of the cardiovascular events. NO-NSAIDs are are able to release NO over prolonged periods of time. The combination of COX inhibition with controlled release of NO yields a series of drugs that exert anti-inflammatory and analgesic activities in a wide range of settings, and have reduced gastrointestinal and cardiorenal toxicity. Recent clinical trials of NO-NSAIDs have provided a ‘proof of concept’ that is completely consistent with preclinical characterization of these compounds. Many pharmaceutical companies have invested millions of dollars in this strategy and although the preclinical and clinical data looked very promising, the FDA outright rejected the first of its kind Naproxcinod, a NO-naproxen drug developed by NicOx. The FDA review recommended further trials in humans to assess the safety on a cardiovascular and gastrointestinal level. NicOx, spent about 10 years and 100 million euros (US$127.6 million) to fund the USA launch of its lead anti-inflammatory drug. This ruling was a major blow to the strategies and pipeline products of big pharma and small biotech. It is currently unclear how industry will respond to further development of this technology.

## S-nitrosothiols

S-nitrosothiols are thio-esters of nitrite with the general structure R-S-N=O (RSNO); naturally occurring examples include S-nitrosocysteine, S-nitrosoglutathione and S-nitrosoalbumin, in which R is an amino acid, polypeptide and protein, respectively. S-nitrosothiols can be synthesized from the reaction between thiols and nitrite via formation of nitrous acid in acidic conditions (pH <3). S-nitrosation is a ubiquitous redox-related modification of cysteine thiol which affects protein structure and function and can elicit cGMP-independent NO signaling [23]. As early as 1981, Ignarro's group demonstrated that the activation of organic nitrates was attributed to reactions with cellular thiols [24], which is several years before making the observation that NO is actually synthesized endogenously in mammalian cells. There is now a large body of literature that implicates S-nitrosothiol (SNO) as an intermediate in nitric oxide dependent and guanylyl cyclase independent signaling processes. S-nitrosothiols may be NO carriers that have vasodilatory properties and may also be integral to the regulation of platelet aggregation. Interest in S-nitrosothiols was heightened by the discovery that NO can react with thiol groups *in vivo* to form S-nitrosothiols such as S-nitrosocysteine and S-nitrosoglutathione (GSNO). In addition, it can exist in the plasma as an S-nitroso adduct of circulating albumin. Consequently, it has been generally assumed that naturally occurring S-nitrosothiols act as *in vivo* storage sites for NO, which can be released upon demand. S-nitrosothiols are susceptible to decomposition by numerous mechanisms, giving rise to NO and the corresponding disulfide. The rate of decomposition is influenced by a number of factors, including metal ions (particularly Cu+), transnitrosation, in other words, the transfer of NO from S-nitrosothiols to other thiols, enzymatic decomposition, photochemical decomposition, thermal decomposition and reaction with ascorbate. S-nitrosothiols are actively metabolized by cells [25]. Some S-nitrosothiols (S-nitrosocysteine, S-nitrosohomocysteine) can be taken up into cells via amino acid transport system L, whereas others (S-nitrosoglutathione, S-nitroso-N-acetylpenicillamine) are not directly transported, but require the presence of cysteine and/or cystine before the nitroso functional group is transported. S-nitrosothiols are biologically active as vasodilators and inhibitors of platelet aggregation. Due to the fact that these low molecular weight nitrosothiols act similarly to EDRF with a longer circulating half-life than NO, make them an ideal candidate for drug development. However, the therapeutic use of S-nitrosothiols such as S-nitroso-N-acetyl-D, L-penicillamine (SNAP) has been limited by their potent vasodilatory effects leading to hypotension.

## NO-releasing nanoparticles

Nanotechnology has emerged as a revolutionary new science. It has the potential to be used as the basis for new, more effective drug delivery systems. Part of the innovation lies in the synthesis of different size particles that may be used for targeted delivery to specific tissue beds or even cancer cells. The use of nanotechnology allows for the effective delivery of a very reactive substance such as NO gas in a way that circumvents many of the limitations associated with other NO delivery systems. Furthermore, precisely and accurately engineered particle constructs can be uniquely manufactured that may eliminate architectural randomness and allow for site-specific targeted distribution. Utilizing specific chemistry in certain hydrogel/glass composites has been shown to provide sustained and predictable release of NO that shows great promise as a new NO delivery system [26]. The use of silicon in nanoparticle constructs has been shown to be effective as a continuous and tunable multistage drug delivery system [27]. This is a very promising strategy to allow for targeted delivery of NO without the unwanted systemic side effects of conventional NO-based therapeutics. Specific technologies will be discussed in much greater detail in the other articles in the issue.

## Summary

In 2006, the total costs of healthcare in the USA exceeded US$2 trillion, or US$6700 per person. This trend continues to increase, estimated to reach US$4 trillion in 2015. Treating chronic diseases such as obesity, diabetes, hypertension, coronary artery disease account for 75% of the nation's annual healthcare costs. According to the American Heart Association, an estimated 81 million people had one or more forms of cardiovascular disease in the USA in 2006, including hypertension, coronary artery disease, myocardial infarction, angina pectoris, stroke and heart failure. Most, if not all, of the chronic conditions mentioned above are the result of a dysfunctional endothelium and inability to produce NO and/or maintain NO homeostasis and signaling. When you consider NO-based technologies may also affect infections, wound healing and targeted cancer treatment, safe and effective NO delivery strategies may constitute the most important advancements in science and medicine in this past two centuries. Understanding and developing new strategies to restore NO homeostasis will have a profound impact on public health and on the healthcare system. Development of NO-based therapies has been slow and largely unsuccessful given the number of FDA-approved NO therapies, the demand for such technologies and the number of disease states that can be affected. Due to the ubiquitous nature of the NO pathway in virtually all biological systems and the precise spatially and temporally controlled regulation and production of NO, it will be challenging to develop targeted NO therapeutics that can recapitulate endogenous endothelial NO production in select tissues. The fact that NO reacts primarily with transition metals and other free radicals, including thiyl radicals to form nitrosothiols as a means to modulate protein structure and function provides finite candidates for drug targets. Most of the disease conditions associated with NO insufficiency is due to dysfunctional NOS or enhanced scavenging of NO once produced or a combination of both. Strategies designed to restore NO production and prevent NO scavenging that allow NO to reach and activate its cellular targets will likely have the most beneficial effects. As we learn more about the downstream targets of NO, effective and specific therapeutics can be developed to activate those pathways that may be able to overcome insufficient NOS production of NO.

## Conclusion

Development of safe and efficacioius nitric oxide technologies will transform disease management. Being able to diagnose or recognize patients at the earliest stages of NO insufficiency prior to the onset and progression of symptoms or disease, and then implement NO therapies will likely lead to effective prevention strategies.

## Future perspective

The nitric oxide field has matured over the past 20 years to a point where both health care practitioners and patients are aware of NO and its effects on health and disease. Due to the ubiquitous nature of NO, technologies that can safely and effectively restore or recapitulate NO based signaling will have broad clinical utility. As we understand more about how the human body regulates and generates NO, dietary strategies may become more safe and effective NO based solutions than NO donating compounds. Harnessing the metabolic activity of nitrate reducing commensal bacteria along with a diet enriched in nitrate and/or nitrite provides a new paradigm for restoring NO in the human body. The interplay between endothelial NO production from NOS and NO generated from nitrate and nitrite will guide researchers and scientists on future development. What is clear is that NO is here to stay and will remain the focus of drug development programs.

**Executive summary**

Development of nitric oxide (NO)-releasing technologies is critically important to many disciplines of science and medicine.
Understanding NO release profiles and targeted delivery will allow for unique therapies from cardiovascular disease, cancer, infections, dermatology and wound healing.
Strategies that can recapitulate endogenous NO biochemistry and restore function will transform medicine and patient care.
